# A single copy integration vector that integrates at an engineered site on the *Staphylococcus aureus *chromosome

**DOI:** 10.1186/1756-0500-5-5

**Published:** 2012-01-05

**Authors:** Mei G Lei, David Cue, Jimena Alba, Jennifer Junecko, Justin W Graham, Chia Y Lee

**Affiliations:** 1 Department of Microbiology and Immunology, University of Arkansas for Medical Sciences, 4301 W. Markham Street, Slot 511, Little Rock, AR 72205, USA

## Abstract

**Background:**

Single-copy integration vectors based upon the site-specific recombination systems of bacteriophage are invaluable tools in the study of bacterial pathogenesis. The utility of such vectors is often limited, however, by the fact that integration often results in the inactivation of bacterial genes or has undesirable effects on gene transcription. The aim of this study is to develop an integration vector that does not have a detectable effect on gene transcription upon integration.

**Findings:**

We have developed a single-copy integration system that enables the cloning vector to integrate at a specific engineered site, within an untranscribed intergenic region, in the chromosome of *Staphylococcus aureus*. This system is based on the lysogenic phage L54a site-specific recombination system in which the L54a phage (*attP*) and chromosome (*attB*) attachment sites, which share an 18-bp identical core sequence, were modified with identical mutations. The integration vector, pLL102, was constructed to contain the modified L54a *attP *site (*attP*2) that was altered at 5 nucleotide positions within the core sequence. In the recipient strain, the similarly modified *attB *site (*attB*2) was inserted in an intergenic region devoid of detectable transcription read-through. Integration of the vector, which is unable to replicate in *S. aureus *extrachromosomally, was achieved by providing the L54a integrase gene in a plasmid in the recipient. We showed that pLL102 integrated specifically at the engineered site rather than at the native L54a *attB *site and that integration did not have a significant effect on transcription of genes immediately upstream or downstream of the integration site.

**Conclusions:**

In this work, we describe an *E. coli*-*S. aureus *shuttle vector that can be used to introduce any cloned gene into the *S. aureus *chromosome at a select site without affecting gene expression. The vector should be useful for genetic manipulation of *S. aureus *and for marking strains for in vivo studies.

## Background

The ability to genetically manipulate bacterial pathogens is essential for the advancement of research on the mechanism of pathogenesis. This holds true for *Staphylococcus aureus*, an important human pathogen that has become one of the most serious infectious agents worldwide [1]. One of the most common genetic manipulations is the complementation test, which is typically carried out using plasmid vectors. In *S. aureus*, most plasmid vectors are multi-copy in nature which could result in overexpression of the complementing gene or could place a heavy metabolic burden on the host bacterium. In order to circumvent these and other problems, we previously developed *S. aureus *vectors that allow cloning of genes in single copy [2,3]. These plasmid vectors are based on the site-specific recombination systems of the lysogenic bacteriophage L54a and Ø11. The vectors can not replicate in *S. aureus*, but because they each carry a bacteriophage *attP *site, they can integrate into the bacterial chromosome at the *attB *site for L54a or Ø11, respectively, in the presence of the bacteriophage integrase (Int) [2,3].

The L54a site-specific recombination system is similar to that of coliphage λ. λ site-specific recombination is mediated by a tyrosine recombinase known as integrase, or Int, protein [4]. In coliphage λ, *attP*, which is much larger than *attB*, provides binding sites for Int and accessory factors whereas the *attB *site only has a binding site for Int [5,6]. λ Int binds to two distinct DNA sites in *attP*, the arm-type and core-type sites. In the recombination reaction, the binding of accessory proteins to *attP *causes DNA bending which allows an Int monomer to bind both the arm site and the half core site simultaneously. During the recombination reaction, a synapsis is formed in which 4 Int monomers bind to 4 *attP *arm sites, 2 *attP *half core sites and 2 *attB *half core sites. The resulting higher order nucleoprotein complex allows the integrase to catalyze the strand exchange between short 7 bp sequences, termed overlap sequences, within the 15 bp core sequence [4,5,7].

In the L54a phage site-specific recombination system, the phage *attP *site and the bacterial *attB *site share an identical 18 bp core sequence [8]. The *attB *site is located near the 5' end of the *geh *gene (SAOUHSC0030), which encodes a glycerol ester hydrolase, and therefore lysogenization by L54a results in a lipase negative phenotype [9]. Inactivation of genes in this manner is known as negative lysogenic conversion [10-12]. We have shown that the L54a *attP *site is between 228-235 bp in length but the L54a *attB *site is less than 27 bp [13]. The *int*-encoded integrase of L54a, like λ Int, has a tyrosine residue that is required for site-specific recombination [9]. Thus, the L54a site-specific recombination system is similar to that of λ.

In addition to the single copy vectors developed by us, other researchers have developed vectors based on the site-specific recombination systems of Ø13 [14], and the staphylococcal pathogenicity island SaPI [15]. All these systems depend on the wild type *attB *site of the respective phage or pathogenicity island. One drawback of using the *S. aureus *phage site-specific recombination systems for construction of single copy vectors is that the *attB *site of most staphylococcal phage is located within an open reading frame (ORF) of a gene [10-12]. As a result, insertion at an *attB *site would interrupt an *S. aureus *gene. This is often undesirable because it could inactivate a gene with an important biological function. In addition, a number of sRNAs have been found in the intergenic regions of the *S. aureus *chromosome which may be affected even if a phage integrates within an intergenic region [16,17]. For the two phage that were the basis of vectors that we previously developed, the L54a *attB *site is within the *geh *gene, whereas the Ø11 *attB *site is within a hypothetical gene that was previously misannotated to be in an intergenic region [2,18]. Furthermore, the *attB *site used for these vectors in some strains may already have an integrated prophage or pathogenicity island. This will interfere with the subsequent transduction from the readily transformable *S aureus *strain RN4220 used as the initial recipient of these vectors. In addition to the site-specific recombination systems mentioned above, a Cre/lox recombination system has also been used as a genetic tool for maker removal in strain construction in *S. aureus *[19].

In this report, we describe the construction of a single-copy integration vector for *S. aureus *based on the L54a site-specific recombination system in which the same mutation was introduced into both *attB *and *attP*. The vector can replicate in *Escherichia coli*, which allows for easy DNA manipulations. In *S. aureus*, the vector integrates with high fidelity into an engineered *attB *site that is devoid of transcription activity.

## Results and discussion

### Identical sequence alteration in L54a *attP *and *attB *affects integration specificity

In the λ site-specific recombination system, a single bp alteration within the 7 bp overlap sequence, in either *attP *or *attB*, drastically reduces the efficiency of recombination. However, the identical change in the partner site restores recombination efficiency [4,20]. Based on this, the λ site-specific recombination system has been developed for recombinant DNA applications (for example, the GATEWAY^® ^cloning system developed by Invitrogen Life Science Technologies).

Since the staphylococcal phage L54a Int-mediated recombination system closely parallels that of λ, we hypothesized that a similar alteration in integration specificity could be achieved in the L54a system [4,20]. This would allow us to develop a single-copy cloning vector that would integrate at a region of our choice with minimal interference on normal biological function of the cells. To this end, we first evaluated 12 intergenic regions for transcriptional activity by RT-PCR. The intergenic region between SAOUHSC00937 and SAOUHSC00938 (ORF designations are according to *S. aureus *strain NCTC8325 annotation) was initially selected for insertion of a mutated *attB *site. We detected very little transcriptional activity in this region in strain 8325-4 cultures grown for 2 h, 4 h or 16 h (data not shown), although two sRNAs are known to be encoded within this region [17].

**Figure 1:**
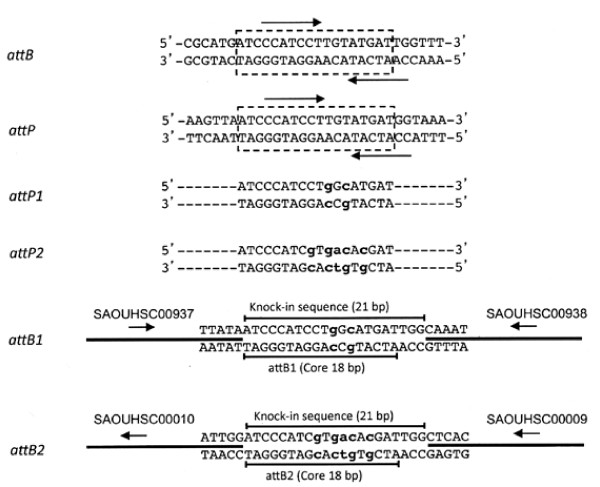
**Sequences of wild type and mutant *att *sites**. *attB*, the wild type *attB *site for L54a integration into the *S. aureus *chromosome. *attP*, the wild type L54a *attP *site for integration at *attB*. The arrows indicate imperfect inverted repeats. The boxed regions signify the 18 bp core region shared between *attP *and *attB*. *attP*1, L54a *attP *site with a 2 bp mutation (indicated in lower case lettering) carried on plasmid pLL3961. The *attP*1 mutations allow for pLL3961 integration into the mutant *attB*1 site. *attP*2, an *attP *site with 5 mutations carried on plasmid pLL102. The *attP*2 mutations allow for pLL102 integration into a mutant *attB*2 site. *attB*1, a mutant *attB *site with the same mutations as *attP*1. The *attB*1 site was placed in the chromosome of RN4220 between bps 911590 and 911591 (NCTC8325 genome) between the SAOUHSC00937 and SAOUHSC00938 orfs. The 21 bp knock in sequence, indicated by the line above the sequence, is bracketed by chromosomal DNA, indicated by the thick lines. The 5' to 3' orientations of SAOUHSC00937 and SAOUHSC00938 are indicated by the arrows. *attB*2, a mutant *attB *site with the same mutations as *attP*2. The *attB*2 site was placed into the bacterial chromosome between the orfs SAOUHSC00009 and SAOUHSC00010 (bps 14208 and 14209). Labeling is the same as for *attB*1.

We then tested whether identical sequence alterations of L54a *attP *and *attB *affect L54a integration specificity. As shown in Figure 1, a pair of 7-bp imperfect inverted repeats, flanking a 3 bp spacer sequence, is located in the L54a *attP *and *attB *sites [8]. The inverted repeats could serve as the binding sites for L54a Int reminiscent of λ Int core binding half sites. The spacer region is similar to the overlap region of λ. Accordingly, we altered 2 bps within the core of the *attP *and *attB *pair by overlapping PCR. The altered *attP *was named *attP*1 (Figure 1). The 1617-bp PCR fragment containing *attP*1 and the adjacent *int *gene was cloned into pCL52.2 resulting in plasmid pLL3961.

The altered *attB *sequence, named *attB*1, was introduced into the bacterial chromosome using the pKOR1 system [21] as previously described [22] using RN4220 DNA as template and primers OU937R9, OU937R10, OU937R11 and OU937R12 (Table 1). The 21-bp *attB*1 fragment was inserted between bp coordinates 911590 and 911591 (*S. aureus *NCTC8325 genome) of the bacterial chromosome. This is within the intergenic region between SAOUHSC00937 and SAOUHSC00938 of strain RN4220, 29 bp downstream of sRNA RsaF [17,23]. The resultant strain, CYL12337, was used as the recipient for integration of pLL3961.

**Table 1:** Primers used in this study

Primer	Sequence
L54aInt1	ggatccacatttagtagctagtactaaaatc

L54aAttP1	ggtttaccatcatgccaggatgggattaacttgtgttaaaaag

L54aAttP2	gttaatcccatcctggcatgatggtaaaccggtcattctct

L54aAttP3	agatctatcgatacggttatatttattcccctac

L54aAttP4	agatctactaaaaggtatctgccctttttctg

L54aAttP5	atcgataatttaggattgtggttattttttgcg

L54aAttP6	ggtttaccatcgtgtcacgatgggattaacttgtgttaaaaag

L54aAttP7	gttaatcccatcgtgacacgatggtaaaccggtcattctct

OU937R3	gaaccaattgaacaagcttgtgaag

OU937R8	ttacttactatttatgaatggccag

OU937R9	gaattcggacaagtttgtacaaaaaagcaggctgtcgctgaagttgcatcaacttgt

OU937attBf	ccaaatttataatttgccaatcatgcc

OU937attBr	gttcgaaaattataatcccatcctggc

OU937R10	gaaaattataatcccatcctggcatgattggcaaattataaatttggtacataatagac

OU937R11	ataatttgccaatcatgccaggatgggattataattttcgaactggttaaattcg

OU937R12	ggatccggaccactttgtacaagaaagctgggtatccatttagctccgattgcttc

OU9R1	ggggacaagtttgtacaaaaaagcaggctcagcaggtagagatacaagagga

OU9R2	tgcattggatcccatcgtgacacgattggctcactattatatttttacagcac

OU9R3	taatagtgagccaatcgtgtcacgatgggatccaatgcacataacaacaataaattaag

OU9R4	ggggaccactttgtacaagaaagctgggtactaaagttttgagacgaagccac

OU9R5	gtaaaaatataatagtgagccaatcgtgtc

OU9R6	tgtgcattggatcccatcgtgac

OU9R7	atgggtggtaaaacacaaatttc

OU9R8	ctcactattatatttttacagcac

OU9R9	ccaatgcacataacaacaataaattaag

OU9R10	catactacatatcaacgaaatcag

SCV1	gcaacaccacataatggttcac

SCV2.1	tgtgccatgataacagcacg

SCV4	acccagtttgtaattccaggag

SCV8	gcacataattgctcacagcca

SAO9F3	aaggtgcgcaattagagcgtgct

SAO9R3	tctgcgttcacaagctgtggtacc

SAO10F3	atgccggtgctgcgcaattt

SAO10R3	ggcgacgccaaa cgtttcgt

PCR was used in order to determine whether pLL3961 had integrated at *attB*1 or the wild type *attB *site. Out of 4 integrants that we tested, 3 were integrated at the correct location, *attB*1 (results not shown). However, in one of the integrants the plasmid had integrated at the wild type L54a *attB *site in the *geh *gene. These results indicate that the altered *att *sites can be recognized by L54a Int but that the specificity of integration is less than perfect. These results also indicate that the size of the L54a *attB *site is less than 21 bp in length, which is shorter than the 27 bp that we previously determined [13].

### Developing pLL102 with high fidelity integration at an engineered *attB *site

**Figure 2:**
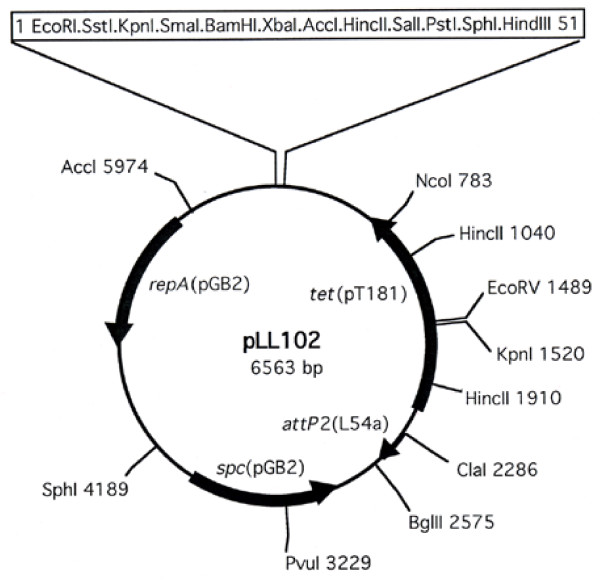
**Map of the integration vector pLL102**. The spectinomycin resistance gene *spc*(pGB2) and replication functions *repA*(pGB2) are both derived from pGB2 [24] and are functional in *E. coli*. The tetracycline resistance gene *tet*(pT181) is expressed in *S. aureus*. The *attP*2 site allows for site-specific recombination between *attP*2 and an engineered *attB*2 site in the *S. aureus *chromosome. The plasmid is maintained in *S. aureus *in a single-copy, integrated form. The plasmid sequence has been deposited to GenBank under the accession number JN639000.

**Figure 3:**
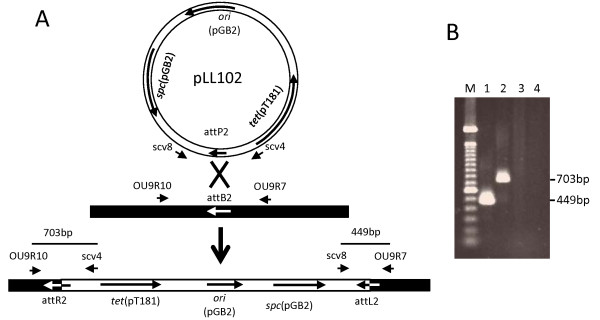
**Integration of pLL102 into an engineered *attB*2 site**. Figure 3A illustrates recombination between the *attP*2 site on pLL102 and the *attB*2 site in the chromosome of CYL12349. The same 5 bp mutations were introduced into the bacteriophage L54a wild type *attP *and *attB *sites to construct *attP*2 and *attB*2. The *attB*2 site was placed in the chromosome (thick solid line) of *S. aureus *strain RN4220 between the orfs for SAOUHSC00009 (*serS*) and SAOUHSC00010. Recombination between *attP*2 and *attB*2 results in integration of pLL102 into the chromosome as illustrated in the lower portion of the figure. The L54a Int protein is provided in trans by pYL112Δ19. Short arrows represent the location of the primers. Figure 3B, Verification of integration via PCR. Genomic DNA was isolated from CYL12376 and used in PCR reactions. Products were analyzed on 1% agarose gels. Lane 1 shows the product from PCR using primers scv8 and OU9R7. Lane 2 shows the product from using primer pairs OU9R10 and scv4. The priming sites and predicted sizes of the PCR products are shown in the lower portion of Figure 3A. Lanes 3 and 4 contain PCR reactions using primer pairs Scv1 and Scv8 and Scv4 and Scv2.1, respectively, to verify that pLL102 did not integrate into the wild type *attB *site that is flanked by Scv1 and Scv2.1 primer sites (not shown [2]). Integration at the wild type *attB *site would generate 1.3 kb and 0.7 kb fragments in lanes 3 and 4, respectively.

The above results suggest that phage integration specificity could be altered by introducing the identical bp changes into the core sequence of *attP *and *attB*, but 2-bp alteration is not enough to ensure high fidelity. Therefore, to increase the specificity, we replaced 5 bp of the *att *core sequence as shown in Figure 1. The new *att *site was named *attP*2. The *attP*2 fragment was then cloned into pCL25 [2], replacing the wild type L54a *attP *fragment, to form pLL102 (Figure 2). Since there is still a low level of transcription at the *attB*1 site described above, we tested additional intergenic regions for insertion of *attB*2 into the chromosome. We found that transcriptional activity in the region between SAOUHSC00009 and SAOUHSC00010 of the 8325-4 chromosome was undetectable at 3 different time points (after 2, 4 and 16 h of culture) by RT-PCR (data not shown). The 21 bp *attB*2 site was then inserted into the intergenic region between G and C at coordinates 14208 and 14209 (NCTC8325 genome) of strain RN4220 (Figure 1) by pKOR1 allele replacement. The resultant strain, CYL12348, was confirmed by PCR. Plasmid pYL112Δ19, which encodes L54a Int [3], was introduced into CYL12348 to generate strain CYL12349. Plasmid pLL102 was then electroporated into CYL12349. Examination of 22 transformants by PCR (Figure 3) showed that all the transformants carried pLL102 integrated at the *attB*2 site. None of the transformants had an insertion at the original L54a *attB *site. These results indicate that pLL102 has a high degree of specificity for the *attB*2 site. The fact that in the *attP*2/*attB*2 pair, the alteration was made in all 3 nucleotides in the spacer region and 2 nucleotides in the mismatched inverted repeat (Figure 1) suggests that these nucleotides are not needed for L54a Int function. These results therefore reaffirm that L54a Int behaves very similarly to that of λ Int suggesting conservation of Int functionality across different species as diverse as *E. coli *and *S. aureus*.

### Integration of pLL102 at the *attB2 *site does not affect transcription of the flanking genes

SAOUHSC00009 (NCTC8325 genome) is annotated as *serS *which encodes seryl-tRNA synthetase, whereas SAOUHSC00010 is a hypothetical branched chain amino acid transporter. The two genes are transcribed in the same orientation with SAOUHSC00009 being upstream of SAOUHSC00010 (Figure 1). The location of the L54a *attB*2 site in the chromosome was selected because we detected no transcription in this region using RT-PCR. The intergenic region is 649 bp in length and there is a rho-independent terminator close to the 3'end of SAOUHSC00009. In addition, there is no known sRNA in this region [16,17]. To avoid interfering with the transcription of the flanking genes, we positioned the *attB*2 insertion site 31 bp downstream of the terminator and 618 upstream of the SAOUHSC00010 start site ATG. Although our RT-PCR experiment did not detect transcripts flanking the insertion site, it is still possible that an artificial insertion at this site could cause alteration in gene expression in the flanking genes. To determine whether the insertion of the *attB*2 site and the subsequent integration of pLL102 affect transcription of the flanking genes, we performed qRT-PCR of the adjacent genes. We found no significant differences in SAOUHSC00009 or SAOUHSC00010 mRNA levels between RN4220, CYL12349 (i.e., RN4220-*attB*2) and CYL12376 (i.e., RN4220 *attB*2(pLL102)) (data not shown) indicating that the insertion of *attB*2 or the integration of pLL102 at the *attB*2 site does not affect expression of the surrounding genes.

## Conclusions

We have developed a single copy integration system that allows insertion of a plasmid vector into the *S. aureus *chromosome at a location devoid of detectable transcription activity. Once a plasmid is inserted into the *attB*2 site, it can then be moved to any other strain by phage transduction. Since there is no detectable transcription activity at the *attB*2 site, the insertion is not likely to alter biological function of the clone. Furthermore, the region flanking the *attB*2 site is highly conserved among all sequenced strains suggesting that moving the integrated plasmid from CYL12349 to other strains is not likely to be hindered by a lack of sequence homology. The system reported here uses a novel, artificial *attB *site, *attB*2, thereby avoiding the disadvantages associated with other bacteriophage derived integration vectors. Additionally, the *attB*2 site is portable and could be inserted into other locations of the chromosome. Thus, our results indicate that plasmid pLL102 is a suitable vector for cloning genes in single copy. In addition, our system can be used to mark a strain with a readily detectable reporter gene, which could be useful for studying pathogenesis in vivo, without affecting gene expression of the marked strains.

## Methods

### Bacterial strains, plasmids and culture conditions

The strains and plasmids used in this study are listed in Table 2. *E. coli *was grown in Luria broth or Luria agar (Difco). *S. aureus *was grown in tryptic soy broth (TSB) or TSB agar (Difco). *E. coli *media were supplemented with 34 μg/ml chloramphenicol, 100 μg/ml penicillin, or 50 μg/ml spectinomycin, as appropriate. *S. aureus *media were supplemented with 10 μg/ml chloramphenicol, or 3 μg/ml tetracycline as appropriate. Recombinant plasmids were initially constructed in *E. coli *then electroporated into *S. aureus *as described [25]. Transduction between *S. arueus *strains was carried out by phage 52A.

**Table 2:** Bacterial strains and plasmids used in this study

Strain or Plasmid	Relevant Characteristics	Reference or Source
*Staphylococcus aureus*		

RN4220	Restriction deficient laboratory strain	[26]

CYL12337	RN4220 *attB*1	This study

CYL12348	RN4220 *attB*2	This study

CYL12349	CYL12348 (pYL112Δ19)	This study

CYL12376	CYL12349 (pLL102)	This study

*E. coli*		

DH5α	Strain for cloning and plasmid maintenance	Invitrogen

XL1-Blue	Strain for cloning and plasmid maintenance	Stratagene

Plasmids		

pCL52.2	Temperature-sensitive cloning vector	[27]

pLL3961	Derivative of pCL52.2 carrying *attP*1 and the L54a *int *gene	This study

pCL25	Carries wild-type *attP *of L54a	[2]

pKOR1	Used for allele replacement in *S. aureus*	[21]

pYL112Δ19	Encodes integrase of phage L54a	[3]

pLL102	Derivative of pCL25 carrying *attP*2	This study

### RT-PCR and qRT-PCR section

RNA for RT-PCR and qRT-PCR was isolated as described [22]. RT-PCR was performed using the Quantitech Reverse Transcription Kit from Qiagen Inc., Valencia, CA. RT-PCR using primers OU9R7, OU9R8, OU9R9 and OU9R10 (Table 1) was performed to probe for transcripts in the intergenic region between SAOUHSC00009 and SAOUHSC00010 using RNA isolated from 4, 8 and 18 h cultures of 8325-4.

To determine whether the insertion of the *attB*2 site and the subsequent integration of pLL102 affected transcription of the flanking genes, we performed qRT-PCR as described [28] of the adjacent genes using primers SAO9F3 and SAO9R3 for SAOUHSC00009 and SAO10F3 and SAO10F3 for SAOUHSC00010. RNA for qRT-PCR was isolated from exponential phase and overnight cultures of strains RN4220, CYL12349 and CYL12376.

### Generation of modified *attP *and *attB*

The *attP*1 and *attB*1 sites were created by altering two bp within the core of *attP *and *attB*, respectively, by overlapping PCR using primers L54aInt1, L54aAttP1, L54aAttP2 and L54aAttP3 (Table 1), and L54a phage DNA as template. The altered *attP *and *attB *were named *attP*1 and *attB*1, respectively (Figure 1). The 1617-bp fragment containing *attP*1 and the adjacent *int *gene was cloned into pCL52.2 resulting in plasmid pLL3961. All PCR-amplified fragments were verified by DNA sequencing.

The altered *attB *sequence, named *attB*1, was introduced into the bacterial chromosome by the pKOR1 system as previously described [21,22] using RN4220 DNA as template and primers OU937R9, OU937R10, OU937R11 and OU937R12 (Table 1). The 21-bp *attB*1 fragment was inserted between bp coordinates 911590 and 911591 (*S. aureus *NCTC8325 genome) of the bacterial chromosome. This is within the intergenic region between SAOUHSC00937 and SAOUHSC00938 of strain RN4220, 29 bp downstream of sRNA RsaF [17,23]. The insertion was verified by PCR using primer pairs OU937attBf/OU937R9 and OU937attBr/OU937R12. The resultant strain, CYL12337, was used as the recipient for integration of pLL3961. The integration was confirmed by PCR using primer pairs OU937R3/L54aAttP3 and OU937R8/L54aAttP3. Primer pairs scv2.1/L54aAttP3 and scv1/L54aAttP4 were used to determine whether a plasmid had integrated at the wild type *attB *site.

The *attP *and *attB *sites with 5 bp substitutions were constructed by overlapping PCR using primers L54aAttP4, L54aAttP5, L54aAttP6 and L54aAttP7 (Table 1). The amplified fragments were verified by DNA sequencing. The new *att *sites were named *attP*2 and *attB*2 (Figure 1). The 289-bp *attP*2 fragment was then cloned into *Cla*I-*Bgl*II-digested pCL25 replacing the 350-bp wild type L54a *attP *fragment to form pLL102 (Figure 2). The 21-bp *attB*2 site was then inserted into the intergenic region between G and C at coordinates 14208 and 14209 (NCTC8325 genome) of strain RN4220 (Figure 1) using the pKOR1 allele replacement vector and primers OU9R1, OU9R2, OU9R3 and OU9R4. The resultant strain, CYL12348, was confirmed by PCR using primer pairs OU9R1/OU9R6 and OU9R4/OU9R5. Plasmid pYL112Δ19, which encodes L54a Int [3], was introduced into CYL12348 by phage 52A transduction and selection for chloramphenicol resistance to generate strain CYL12349. Plasmid pLL102 was then electroporated into CYL12349 and transformants were selected with 3 μg/ml tetracycline.

## Competing interests

The authors declare that they have no competing interests.

## Authors' contributions

MGL, DC and CYL designed the research and wrote the manuscript. MGL carried out chromosome site selection experiments. DC carried out gene expression experiments. JA performed technical experiments in plasmid and strain construction. JJ and JWG carried out strain construction and integration experiments. All authors read and approved the final manuscript.
